# Coding-Complete Genome Sequences of Two SARS-CoV-2 Isolates from Early Manifestations of COVID-19 in Israel

**DOI:** 10.1128/MRA.00677-20

**Published:** 2020-07-09

**Authors:** Inbar Cohen-Gihon, Ofir Israeli, Ohad Shifman, Dana Stein, Hagit Achdout, Shay Weiss, Michal Mandelboim, Oran Erster, Gili Regev-Yochay, Gad Segal, Shmuel Yitzhaki, Shmuel C. Shapira, Adi Beth-Din, Anat Zvi

**Affiliations:** a Department of Biochemistry and Molecular Genetics, Israel Institute for Biological Research, Ness-Ziona, Israel; b Department of Infectious Diseases, Israel Institute for Biological Research, Ness-Ziona, Israel; c Central Virology Laboratory, Israel Ministry of Health, Chaim Sheba Medical Center, Tel-Hashomer, Ramat-Gan, Israel; d Chaim Sheba Medical Center, Tel-Hashomer, Ramat-Gan, Israel; e Israel Institute for Biological Research, Ness-Ziona, Israel; Queens College

## Abstract

We announce the genome sequences of two strains of severe acute respiratory syndrome coronavirus 2 (SARS-CoV-2) isolated in Israel, one imported by a traveler who returned from Japan and the second strain collected from a patient infected by a traveler returning from Italy. The sequences obtained are valuable as early manifestations for future follow-up of the local spread of the virus in Israel.

## ANNOUNCEMENT

Since its first occurrence in China, in December 2019 (1, 2), severe acute respiratory syndrome coronavirus 2 (SARS-CoV-2), belonging to the *Coronaviridae* family and the *Betacoronavirus* genus, has been extensively spreading to the rest of the world. The first cases were imported and identified in Israel in late February 2020 with the arrival of passengers from the *Diamond Princess* cruise ship. Since then, over 20,000 cases (as of June 2020) have been reported in Israel.

In this report, we describe the sequencing of two strains isolated from two subjects, one traveler who returned to Israel from Japan and a patient who was infected by a traveler returning from Italy. These subjects are among the first confirmed coronavirus disease 2019 (COVID-19) cases in Israel. This study was reviewed and approved by the Institutional Review Board at the Chaim Sheba Medical Center. The two patients showed mild symptoms and recovered within a few weeks. The patients were initially identified as positive for COVID-19 by reverse transcriptase quantitative PCR (RT-qPCR). The samples were collected directly from nasopharyngeal swabs. Tubes containing the swabs were vortexed for 2 min, and samples from the transfer buffer were overlaid on Vero E6 cell monolayers (ATCC CRL-1586, 37°C, 5% CO_2_). After 4 days, RNA was purified from 140 μl of the cell culture supernatant using a QIAamp viral RNA minikit (Qiagen) according to the manufacturer’s protocol. A SMARTer stranded total RNA-Seq kit v2-pico input mammalian (TaKaRa Bio) was used for library construction prior to sequencing. Whole-genome paired-end (PE) sequencing was conducted on a MiSeq instrument using the 300-PE v2 kit (Illumina) with a read length of 150 nucleotides. This produced 1,522,094 and 7,149,870 reads for the Japanese and Italian strains (here, denoted ISR_JP0320 and ISR_IT0320), respectively. FastQC (https://www.bioinformatics.babraham.ac.uk/projects/fastqc) with default settings was used for quality control of the data. Trimming and removal of low-quality reads was performed using Trim Galore! v0.6.3 (http://www.bioinformatics.babraham.ac.uk/projects/trim_galore/) with default settings. Reads originating from the Vero E6 host cells were filtered out using Bowtie 2 (3) with default parameters, resulting in 20,218 and 226,488 reads for the ISR_JP0320 and ISR_IT0320 strains, respectively. Genome assembly was performed using SPAdes v3.13.0 (4) with default parameters and produced a single contig for each strain, consisting of 29,851 and 29,870 nucleotides for the ISR_JP0320 and ISR_IT0320 strains, respectively. The overall G+C contents of the sequences were 38.02% and 38.05% for the ISR_JP0320 and ISR_IT0320 strains, respectively. The average coverages were 150× and 932× for the ISR_JP0320 and ISR_IT0320 strains, respectively. Mapping of the resulting reads was conducted using Bowtie 2 (3) against the reference Wuhan strain (GenBank accession number NC_045512) and revealed the existence of 3 mutations for the ISR_JP0320 strain (T5137→C, G11083→T, and G15438→A) and 8 mutations for the ISR_IT0320 strain (C241→T, C313→T, C3037→T, C14408→T, A23403→G, G28881→A, G28882→A, and G28883→C). The Italian strain harbors a documented replacement of D614→G in the spike protein, a replacement which is typical of European strains (https://nextstrain.org/ncov), compared with the strain imported from Japan.

**FIG 1:**
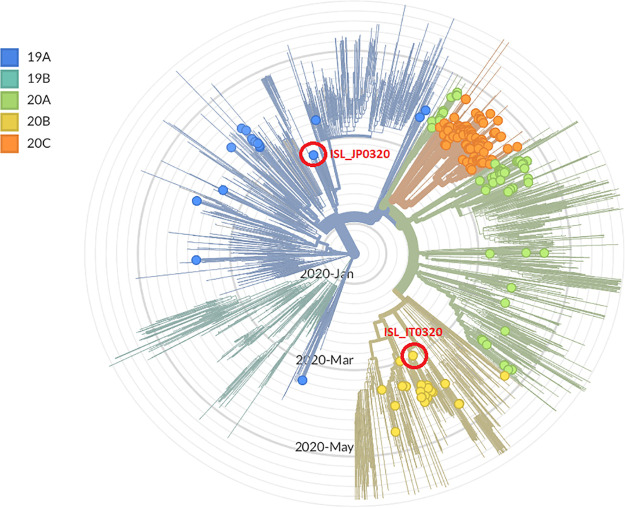
Phylogenetic tree displaying sequenced SARS-CoV-2 strains, according to the designated clades of the virus. The tree was generated by the Nextstrain site using the bioinformatics toolkit Augur, which implements FastTree, which infers approximately maximum-likelihood phylogenetic trees from alignments of nucleotide or protein sequences (5, 7). All strains are colored by their assigned clade, according to the nomenclature established recently in reference 6. Background, strains of the Asia data set available in Nextstrain; dots, all strains collected in Israel; circled in red, the two strains documented in the current study, ISR_JP0320 and ISR_IT0320.

The radial presentation of a phylogenetic tree generated by Nextstrain (5) is presented in Fig. 1 for all the Asian strains available to date in the Nextstrain data set. The phylogeny is rooted relative to early samples from Wuhan, China. The tree displays the two sequenced strains in the context of over 200 sequences of strains collected in Israel in the past 2 months, colored by the different clades designated for SARS-CoV-2 (6). The ISR_JP0320 strain was identified as belonging to clade 19A (Asia), while the ISR_IT0320 strain was identified as belonging to the 20B clade (Europe). These strain sequences represent an early manifestation of the virus in Israel and are therefore important as starting points for studying the epidemiology and evolution of the virus and its circulation in Israel.

### Data availability.

These sequences have been deposited at the GISAID EpiCoV coronavirus SARS-CoV-2 platform database under identifiers EPI_ISL_419211 and EPI_ISL_419210 and in the NCBI GenBank database under the accession numbers MT276597 and MT276598. The raw reads have been submitted to the NCBI Sequence Read Archive under the accession numbers PRJNA635017 and PRJNA626526.
